# Supplementary material for: Trends in Health-Risk Behaviors and Psychological Distress among Australian First-Year University Students
Source: Int J Environ Res Public Health. 2024 May 14;21(5):620. doi: 10.3390/ijerph21050620 (PMC11121384; doi:10.3390/ijerph21050620)
Supplement: Supplementary file 1 [file ijerph-21-00620-s001.zip › ijerph-2952375-supplementary.pdf]

Supplementary Table S1. The proportion of first year, undergraduate University of Newcastle students aged 17-24 years meeting and not meeting the guidelines for each of the health risk behaviour and mental health outcomes, and the number without a result (missing), for each of the survey years (2016, 2017, 2019, 2020).

Health behaviour		*2016 (N = 1300)*	*2017 (N = 484)*	*2019 (N = 456)*	*2020 (N = 571)*
Meeting Fruit Consumption Guidelines	Not meeting	562 (43%)	220 (45%)	233 (51%)	279 (49%)
	Meeting	738 (57%)	264 (55%)	223 (49%)	292 (51%)
	Missing	0	0	0	0
Meeting Vegetable Consumption Guidelines	Not meeting	1196 (92%)	451 (93%)	426 (93%)	522 (91%)
	Meeting	104 (8.0%)	33 (6.8%)	30 (6.6%)	49 (8.6%)
	Missing	0	0	0	0
Frequency of Breakfast Consumption	Missing	0	0	0	0
	Less than everyday	585 (45%)	225 (46%)	235 (52%)	321 (56%)
	Everyday	715 (55%)	259 (54%)	221 (48%)	250 (44%)
Frequency of Takeaway Consumption	Missing	0	0	0	0
	Less than 1-2 times/wk	832 (64%)	296 (61%)	288 (63%)	328 (57%)
	1-2 times/wk or more	468 (36%)	188 (39%)	168 (37%)	243 (43%)
Performing Sufficient Physical Activity	Missing	9	24	11	28
	Insufficient physical activity	306 (24%)	150 (33%)	119 (27%)	123 (23%)
	Sufficient physical activity	985 (76%)	310 (67%)	326 (73%)	420 (77%)
Sitting Time	Missing	0	0	0	0
	< 8 hours/day	814 (63%)	274 (57%)	307 (67%)	362 (63%)
	> or = 8 hours/day	486 (37%)	210 (43%)	149 (33%)	209 (37%)
Tobacco Smoking Use	Missing	0	0	0	0
	Non-smoker	1208 (93%)	462 (95%)	419 (92%)	534 (94%)
	Smoker	92 (7.1%)	22 (4.5%)	37 (8.1%)	37 (6.5%)
Alcohol Single Occasion Risk	Missing	0	0	0	0
	Low	792 (61%)	265 (55%)	270 (59%)	369 (65%)
	High	508 (39%)	219 (45%)	186 (41%)	202 (35%)
Lifetime Drug Use	Missing	61	8	0	0
	Drug use	390 (31%)	173 (36%)	186 (41%)	199 (35%)
	No drug use	849 (69%)	303 (64%)	270 (59%)	372 (65%)
Sleep	Missing	0	0	0	0

Health behaviour		*2016 (N = 1300)*	*2017 (N = 484)*	*2019 (N = 456)*	*2020 (N = 571)*
	Not meeting recommendations (under or over)	375 (29%)	126 (26%)	92 (20%)	149 (26%)
	Meeting recommendations	925 (71%)	358 (74%)	364 (80%)	422 (74%)
Kessler Psychological Distress	Missing	39	22	13	19
	Low	817 (65%)	226 (49%)	206 (47%)	249 (45%)
	High	444 (35%)	236 (51%)	237 (53%)	303 (55%)
